# The need for future coronary access in older medicare beneficiaries following transcatheter aortic-valve replacement

**DOI:** 10.1007/s12928-025-01171-0

**Published:** 2025-07-18

**Authors:** Christopher Brown, Michael Ryan, Marcella Kelley, Christin Thompson, Candace Gunnarsson, James Hermiller

**Affiliations:** 1 Swedish Heart and Vascular Institute, 550 17th Ave Suite 680, Seattle, WA 98122 USA; 2 MPR Consulting, Cincinnati, OH USA; 3 Edwards Lifesciences, Irvine, CA USA; 4 Gunnarsson Consulting, Jupiter, FL USA; 5 Ascension St. Vincent’s Heart Center of Indiana, Indianapolis, IN USA

**Keywords:** Coronary access, TAVR, Medicare beneficiaries

## Abstract

**Background:**

While approximately 17% of patients less than 80 years old require coronary access in the 7 years following their TAVR, the need for coronary access among older TAVR patients is unknown. Methods: We examined the percentage of Medicare beneficiaries aged 80–90 years that require coronary access [percutaneous coronary intervention (PCI) or angiogram] in the 8 years following their TAVR using data from the Medicare 5% Standard Analytic File (2011–2021). The need for coronary access in older patients was estimated for all TAVRs, TAVR patients with and without a history of PCI, and TAVR patients with and without coronary artery disease (CAD) using time-to-event models adjusted for age, sex, race, region, ECI score, concomitant CABG, CAD, PCI, and current or recent smoker status. Multivariate log-gamma regressions were used to estimate the total cost of hospitalizations requiring coronary access post-TAVR. Results: A total of 6845 patients met inclusion criteria. The incidence rates for undergoing PCI or angiogram at 1, 3, 5, and 8 years were 1.9%, 4.0%, 5.5%, and 6.3%, respectively. TAVR patients with PCI demonstrated higher rates of coronary intervention compared to those without PCI (10.2% vs. 6.2% at 8 years, respectively). Similarly, TAVR patients with a prior CAD diagnosis exhibited increased rates of coronary intervention compared to those without a prior CAD diagnosis (7.4% vs. 2.1% at 8 years, respectively). The mean adjusted cost of hospitalizations requiring coronary access was $30,170 [95% Confidence Interval: $27,865-$32,665]. Conclusions: Approximately 6.8% of older TAVR patients require coronary access in the 8 years following their index procedure. The presence of a prior PCI or CAD diagnosis is associated with an increased requirement for subsequent coronary access.

**Graphical abstract:**

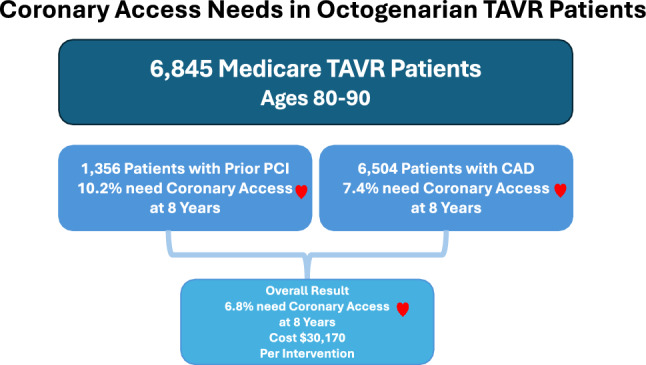

## Introduction

Coronary artery disease is the most common comorbid condition affecting outcomes in patients with AS post-aortic-valve replacement (AVR) [1–6]. Historically, the standard treatment pathway for patients with both severe AS and CAD has been surgical AVR (SAVR) and revascularization via concomitant coronary artery bypass grafting [3, 5, 7, 8]. The advent of transcatheter aortic-valve replacement (TAVR), has transformed the therapy of severe AS, as more patients now receive TAVR over isolated SAVR in the United States [9]. Despite the frequency and importance of concomitant coronary artery disease, the optimal strategy for managing coexisting CAD before and after TAVR remains a fundamental unanswered question [3, 5, 10, 11]. Some advocate for pre-TAVR percutaneous coronary intervention, while others manage it expectantly [12].

Current guidelines provide limited direction on the management of CAD in TAVR candidates. The 2020 ACC/AHA Guideline for the Management of Valvular Heart Disease offers a Class IIb recommendation (Level of Evidence C) for revascularization before TAVR in patients with significant CAD, acknowledging the limited evidence supporting this approach [13]. Recent randomized-controlled trials have attempted to address this knowledge gap. The ACTIVATION trial randomized 235 patients to either prophylactic PCI before TAVR or no PCI, finding no significant difference in 1-year all-cause mortality, stroke, myocardial infarction, or repeat revascularization [14].

As indications for TAVR expand to include a younger population of low-risk surgical patients, [15–20] the need to understand the frequency with which CABG or PCI is required post-TAVR was increasingly becoming more important. As such, Hermiller and colleagues (2021) analyzed Medicare patients < 80 years of age at the time of their aortic-valve replacement (AVR) between 2011 and 2018 and found that 2.5% of patients at 1-year post-index and 17% at 7 years would need coronary access. For TAVR patients with CAD at baseline, 22% of patients required coronary access at 3 years versus 7% for those without CAD [21].

There is less evidence regarding the need for coronary access in older TAVR patients. Approximately 75% of severe symptomatic AS patients aged 80 years or older do not receive AVR, despite octogenarians experiencing as favorable of TAVR outcomes as septuagenarians [22, 23]. As efforts to increase the treatment of older AS patients continue, understanding the need for future coronary access can inform initial procedural planning and potentially valve choice.

The objective of this study is to build upon Hermiller and colleagues (2021) publication and to estimate the percentage of Medicare patients ages 80–90 that will need coronary access (for PCI or an angiogram) over an 8-year time horizon following their index TAVR for the following cohorts: (1) All TAVRs, (2) TAVR patients with and without a record of PCI at 12 months baseline or on Index, and (3) TAVR patients with and without a record of CAD at 12 months baseline or on Index. Additionally, we aimed to quantify the economic burden of post-TAVR coronary interventions to inform healthcare resource planning.

## Methods

### Data source

This study utilized data from the Medicare Limited Dataset (LDS) 5% Standard Analytic File (SAF) for the years 2011–2021. The LDS SAFs are derived from Medicare fee-for-service (FFS) claims data and contains detailed, beneficiary-level information for healthcare services and utilization covered by Medicare Parts A and B. The 5% SAF is a randomized sub-sample that includes claims data provided by physicians and durable medical equipment suppliers in addition to data from hospitals (inpatient and outpatient), skilled nursing facilities, home health agencies, freestanding clinics, and ambulatory surgical centers. The database allows researchers to link utilization for individual beneficiaries over time and across providers; and, comprehensive claims data submitted by providers include, but are not limited to, the following: annual demographic and enrollment information; from and through dates; admission and discharge dates; the source of admission and discharge destination (including death) for institutional providers; diagnosis and procedure codes; provider identity; Medicare program payments; and beneficiary responsibility. Cost data are derived from facility-specific cost-to-charge ratios based on charges in the dataset.

This study did not require institutional review board (IRB) approval, because all Medicare LDS data are encrypted to eliminate any direct patient identifiers, ensuring Health Insurance Portability and Accountability Act (HIPAA) compliance.

### Inclusion criteria and variables of interest

Medicare patients with a record of a TAVR between January 1, 2011, and December 31, 2021, were included for analysis. Patients were required to be 80–90 years of age at the time of their TAVR admission. The primary outcome of interest was the percentage of patients who required coronary access after a TAVR over an 8-year time horizon. Sub-populations of interest included: TAVR patients with and without a record of PCI at 12 months baseline or on Index, and TAVR patients with and without a record of CAD at 12 months baseline or on Index. The secondary outcome of interest was the total cost of the first hospitalization requiring coronary access post-TAVR.

Patient demographic information included age, sex, race, region, a record of PCI, comorbid conditions, including CAD, current or history of smoking, and the 31 categories of comorbidities associated with mortality as measured by the Elixhauser Comorbidity Index (ECI) [24].

### Statistical analysis

Demographic information, ECI score, previous CAD, smoking, or PCI anytime were summarized for all AVR patients and by the sub-populations of interest.

Time-to-event analyses were conducted using competing risk models to estimate the primary outcome, the risk of PCI, or an angiogram each year for over 8 years following a patient’s index AVR procedure. The overall AVR multivariable time-to-event model adjusted for age, sex, race, region, ECI score, concomitant CABG, CAD, PCI, and current or recent smoker status. Patients were censored if they died or dropped out of the database.

Log-gamma regressions were used to estimate the total cost of hospitalizations requiring coronary access post-TAVR, adjusted for the same covariates used in the primary model. For patients with multiple coronary access hospitalizations following TAVR, the secondary analysis was limited to the patient’s first hospitalization. All statistical analyses were performed using SAS software, version 9.4 (SAS Institute Inc., Cary, NC, USA).

## Results

**Fig. 1:**
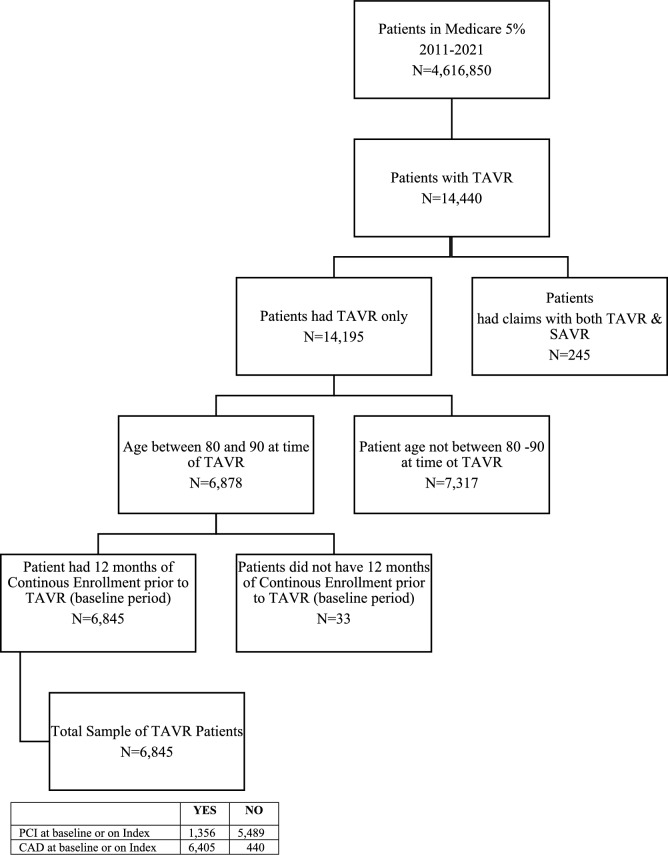
Attrition diagram

A total of 14,440 patients in the Medicare 5% SAF underwent TAVR between January 1, 2011, and December 31, 2021, with 245 (1.70%) patients excluded due to a record of both TAVR and SAVR on index. The final sample of patients that were between the age of 80 and 90 during their index TAVR and continuously enrolled in the year prior is 6845 patients. Sample sizes for our sub-populations are as follows: TAVR patients with and without a record of PCI at 12 months baseline or on Index (1356; 5,489) and TAVR patients with and without a record of CAD at 12 months baseline or on Index (6405; 440). See Fig. 1 for complete attrition diagram.

Table 1 provides patient characteristics for all TAVRs and sub-populations of interest. The mean (SD) age of the study cohort was 84.45 (2.96) years. The majority of patients were male (52.5%), Caucasian (94.4%), had a record of CAD (93.6%), and did not have a record of PCI (80.2%). The most frequent region was the South (34.1%). The mean (SD) ECI score for the overall study cohort was 8.03 (3.75), and slightly higher in the sub-populations with a record of PCI (8.92) or CAD (8.19). Approximately half of the sample had a history of smoking anytime or was a current smoker prior to their AVR.

**Table 1:** Patient characteristics

	TAVR	TAVR W/PCI Pre/Index	TAVR WO/PCI Pre/Index	TAVR W/CAD	TAVR WO/CAD
Total patients	6845	1356	5489	6405	440
Age					
Mean (SD)	84.45 (2.96)	84.50 (2.94)	84.43 (2.97)	84.45 (2.96)	84.39 (3.02)
Gender					
Female	3250 (47.48%)	557 (41.08%)	2693 (49.06%)	2926 (45.68%)	324 (73.64%)
Male	3595 (52.52%)	799 (58.92%)	2796 (50.94%)	3479 (54.32%)	116 (26.36%)
Race					
Caucasian	6458 (94.35%)	1282 (94.54%)	5176 (94.30%)	6049 (94.44%)	409 (92.95%)
Black	186 (2.72%)	34 (2.51%)	152 (2.77%)	161 (2.51%)	25 (5.68%)
Hispanic	54 (0.79%)	* (*)	45 (0.82%)	52 (0.81%)	* (*)
Asian	54 (0.79%)	13 (0.96%)	41 (0.75%)	53 (0.83%)	* (*)
Other/unknown	93 (1.36%)	18 (1.33%)	75 (1.37%)	90 (1.41%)	* (*)
Region					
Midwest	1,611 (23.54%)	353 (26.03%)	1,258 (22.92%)	1,517 (23.68%)	94 (21.36%)
Northeast	1644 (24.02%)	270 (19.91%)	1374 (25.03%)	1547 (24.15%)	97 (22.05%)
South	2,332 (34.07%)	476 (35.10%)	1,856 (33.81%)	2,198 (34.32%)	134 (30.45%)
West	1240 (18.12%)	256 (18.88%)	984 (17.93%)	1131 (17.66%)	109 (24.77%)
Missing/unknown	18 (0.26%)	* (*)	17 (0.31%)	12 (0.19%)	* (*)
Elixhauser score					
Mean (SD)	8.03 (3.75)	8.92 (3.33)	7.81 (3.81)	8.19 (3.68)	5.66 (3.87)
Other comorbidities					
CAD pre or on index	6405 (93.57%)	1356 (100%)	5049 (91.98%)	6405 (100%)	* (*)
Smoker Current or anytime past	3468 (50.66%)	729 (53.76%)	2739 (49.90%)	3310 (51.68%)	158 (35.91%)

*AVR* aortic-valve replacement, *TAVR* transcatheter AVR, *SAVR* surgical AVR, *CABG* coronary artery bypass graft, *ECI* elixhauser comorbidity index, *CAD* coronary artery disease

**Fig. 2:**
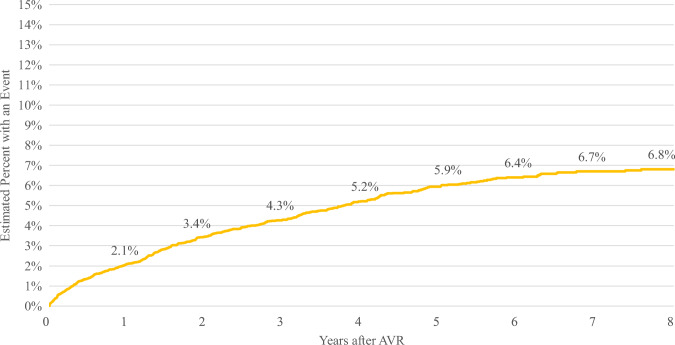
Time to coronary access for all TAVR patients

Table 2 summarizes the outcomes from the multivariable competing risk regression analyses for time to PCI or angiogram at the following intervals: 1, 3, 5, and 8 years following index for all patients undergoing TAVR, segmented by specific cohorts under investigation. Across the entire TAVR patient group, the incidence rates for undergoing PCI or angiogram at the aforementioned time points were 2.1%, 4.3%, 5.9%, and 6.8%, respectively (refer to Table 2 and Fig. 2).

**Table 2:** Summary of regression results (time to PCI or angiogram)

Cohort	1 year	3 years	5 years	8 years
TAVRS	2.1%	4.3%	5.9%	6.8%
TAVRS W/PCI	3.4%	6.8%	9.1%	10.2%
TAVRS WO/PCI	2.0%	4.1%	5.5%	6.2%
TAVRS W/CAD	2.4%	4.8%	6.5%	7.4%
TAVRS WO/CAD	0.7%	1.4%	1.9%	2.1%

**Fig. 3:**
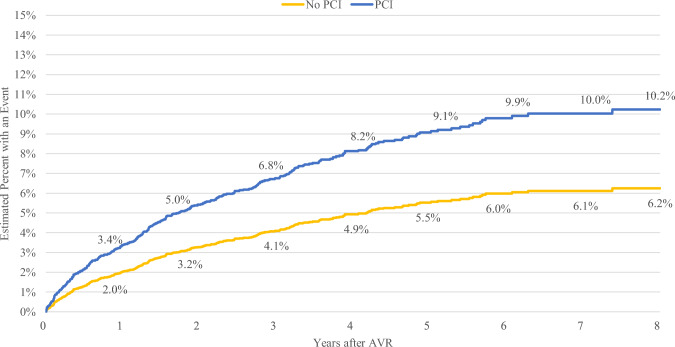
Time to coronary access for TAVR with and without PCI

**Fig. 4:**
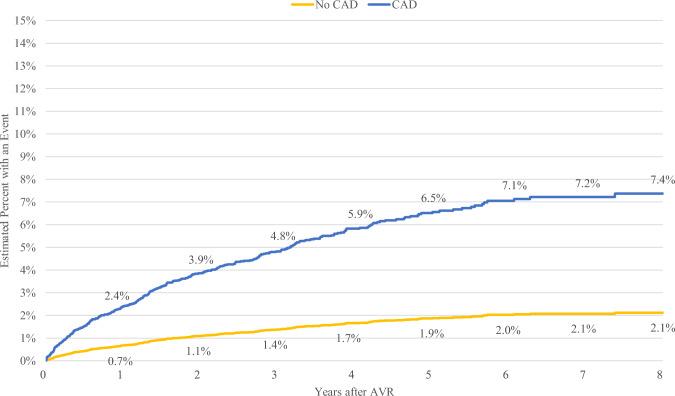
Time to coronary access for TAVR with and without CAD

For TAVR recipients, the presence of a prior PCI or CAD diagnosis was associated with an increased requirement for subsequent coronary access. Specifically, TAVR patients with PCI demonstrated higher rates of coronary intervention at 1, 3, 5, and 8 years post-index compared to those without PCI, with percentages at 3.4% vs. 2.0%, 6.8% vs. 4.1%, 9.1% vs. 5.5%, and 10.2% vs. 6.2%, respectively (refer to Table 2 and Fig. 3). Similarly, TAVR patients with a prior CAD diagnosis exhibited increased rates of coronary intervention compared to those without a prior CAD diagnosis at these intervals: 2.4% vs. 0.7% at 1 year, 4.8% vs. 1.4% at 3 years, 6.5% vs. 1.9% at 5 years, and 7.4% vs. 2.1% at 8 years (see Table 2 and Fig. 4).

In the secondary analysis of cost outcomes, 273 patients were included. The mean adjusted cost of hospitalizations requiring coronary access was $30,170 [95% Confidence Interval: $27,865-$32,665].

## Discussion

In a contemporary cohort of 6845 patients, approximately 6.8% of patients who receive a TAVR at age 80–90 require a PCI or an angiogram within 8 years. Rates of coronary access are higher for patients that had a record of PCI at baseline (10.2% versus 6.2%) and those with a record of CAD at baseline (7.4% versus 2.1%). The mean cost of a hospitalization requiring coronary access following TAVR is approximately $30,000.

Our findings demonstrate the impact of advanced age on post-AVR coronary access needs. Compared to the younger Medicare cohort (< 80 years, mean age 70.6) studied by Hermiller et al. [21], our octogenarian population showed substantially lower rates of coronary access: 2.1% vs 2.5% at 1 year and 6.8% vs 17% at 7–8 years. This age-related difference was particularly pronounced in high-risk subgroups. While 28% of younger TAVR patients with baseline PCI required coronary access at 4.5 years, our older cohort with baseline PCI had rates of 10.2% at 8 years. Similarly, younger TAVR patients with CAD showed 22% coronary access rates at 3 years compared to 4.8% in our octogenarian CAD cohort at the same timepoint. These substantial differences likely reflect competing mortality risks, more conservative treatment approaches in very elderly patients, and shorter life expectancy that limits the time window for coronary events requiring intervention.

The consensus across studies that have evaluated PCI and angiograms after an AVR is that in both SAVR and TAVR coronary access is possible and relatively safe, but can be challenging depending on patient-prosthesis geometric relationships [6, 10, 25–40].

While our study demonstrates the frequency of coronary access needs post-TAVR in older patients, it is important to consider how valve selection impacts reaccess feasibility. Coronary reaccess challenges vary significantly by valve design and implantation technique. Self-expanding valves with supra-annular leaflet positioning (e.g., Medtronic's CoreValve/Evolut) present distinct challenges compared to balloon-expandable valves (e.g., Edward’s SAPIEN) with their intra-annular leaflet configuration and shorter frame height. Technical factors that influence coronary reaccess include commissural alignment relative to coronary ostia, valve frame height in relation to coronary ostial height, strut configuration and cell size, and leaflet design characteristics [35–37]. These considerations have particular relevance in our study population of octogenarians and nonagenarians, who may present with more complex anatomical features such as increased aortic angulation, extensive calcification patterns, and variable coronary heights. Given our findings that patients with prior CAD or PCI demonstrate substantially higher rates of subsequent coronary interventions (7.4% and 10.2% at 8 years, respectively), consideration of valve design and its impact on future coronary access may be particularly warranted in these higher-risk subgroups.

One of the primary importances of coronary access post-TAVR question is cost. The cost implications of hospitalizations requiring coronary access post-TAVR were not previously well understood. Our results indicate that coronary access post-TAVR resembles costs of PCI in more severe cardiac patients. The estimates reported in our sample are lower than the costs reported for PCI for coronary syndrome ($23,541) and similar to the previously reported average cost of PCI or coronary angiograms in patients hospitalized for heart failure ($34,848 and $26,282, respectively) [39, 40]. From a healthcare planning perspective, these findings suggest that approximately 1 in 15 octogenarian TAVR patients will require a costly coronary intervention, with higher rates in those with established coronary disease. This information can help inform patient and family discussions about potential long-term care needs and assist healthcare systems in resource allocation planning for post-TAVR follow-up care.

Despite the importance of CAD post-AVR, scant data exist regarding the need for coronary access post-AVR, especially in older patients aged 80–90 and understanding of post-TAVR PCI frequency helps inform procedure and potentially valve choice. Considerations to allow for future coronary access are critical for TAVR patients 80–90 years old.

### Limitations

Inherent limitations exist in this study that need to be acknowledged. First, coding errors, like the under-coding of non-billable events, are possible. Additionally, this analysis was performed on Medicare patients, who are older and are more likely to have advanced disease and comorbidities. However, severe AS and CAD are more prevalent in older populations, and we, therefore, believe that Medicare patients are largely representative of the patients undergoing AVRs in clinical practice. Additionally, while statistical models controlled for several factors, it was not possible to account for all variables that may have affected our projections.

Additionally, administrative claims data do not capture specific valve types or manufacturer information. Valve design characteristics significantly impact coronary reaccess feasibility, and operator valve selection may already account for anticipated future coronary intervention needs, potentially affecting our estimates.

## Conclusions

Among Medicare beneficiaries aged 80 to 90 undergoing TAVR, approximately 6.8% will require coronary access (PCI or an angiogram) within 8 years. Patients with a history of CAD or PCI had a higher need for coronary access at 8 years: 7.4% and 10.2%, respectively.

## Abbreviations

AS: Aortic stenosis
AVR: Aortic-valve replacement
SAVR: Surgical aortic-valve replacement
TAVR: Transcatheter aortic-valve replacement
ECI: Elixhauser comorbidity index
ED: Emergency department
SNF: Skilled nursing facility
